# Supplementary material for: Age-related reorganization of functional network architecture in semantic cognition
Source: Cereb Cortex. 2022 Oct 3;33(8):4886–903. doi: 10.1093/cercor/bhac387 (PMC10110455; doi:10.1093/cercor/bhac387)
Supplement: SupplementaryMaterials_rev1_bhac387 [file supplementarymaterials_rev1_bhac387.zip › SupplementaryMaterials_rev1_bhac387.docx]

**Supplementary Materials**

**Age-related reorganization of functional network architecture for language processing**

Sandra Martin, Kathleen A. Williams, Dorothee Saur, Gesa Hartwigsen

**Contents**

[Supplementary Methods 1](#_Toc112057722)

[Figure S1 1](#_Toc112057723)

[Figure S2 2](#_Toc112057724)

[Figure S3 3](#_Toc112057725)

[Figure S4 8](#_Toc112057726)

[Figure S5 9](#_Toc112057727)

[Figure S6 10](#_Toc112057728)

[Supplementary Results 11](#_Toc112057729)

[Figure S7 11](#_Toc112057730)

[Table S1 12](#_Toc112057731)

[Table S2. 13](#_Toc112057732)

[Table S3. 14](#_Toc112057733)

[Table S4. 18](#_Toc112057734)

[Table S5. 19](#_Toc112057735)

[Table S6. 20](#_Toc112057736)

[Table S7 20](#_Toc112057737)

[Table S8 21](#_Toc112057738)

[Table S9 21](#_Toc112057739)

[Table S10 22](#_Toc112057740)

[Table S11 22](#_Toc112057741)

[Table S12 24](#_Toc112057742)

[Table S13 24](#_Toc112057743)

[Table S14 25](#_Toc112057744)

# Supplementary Methods

## Figure S1


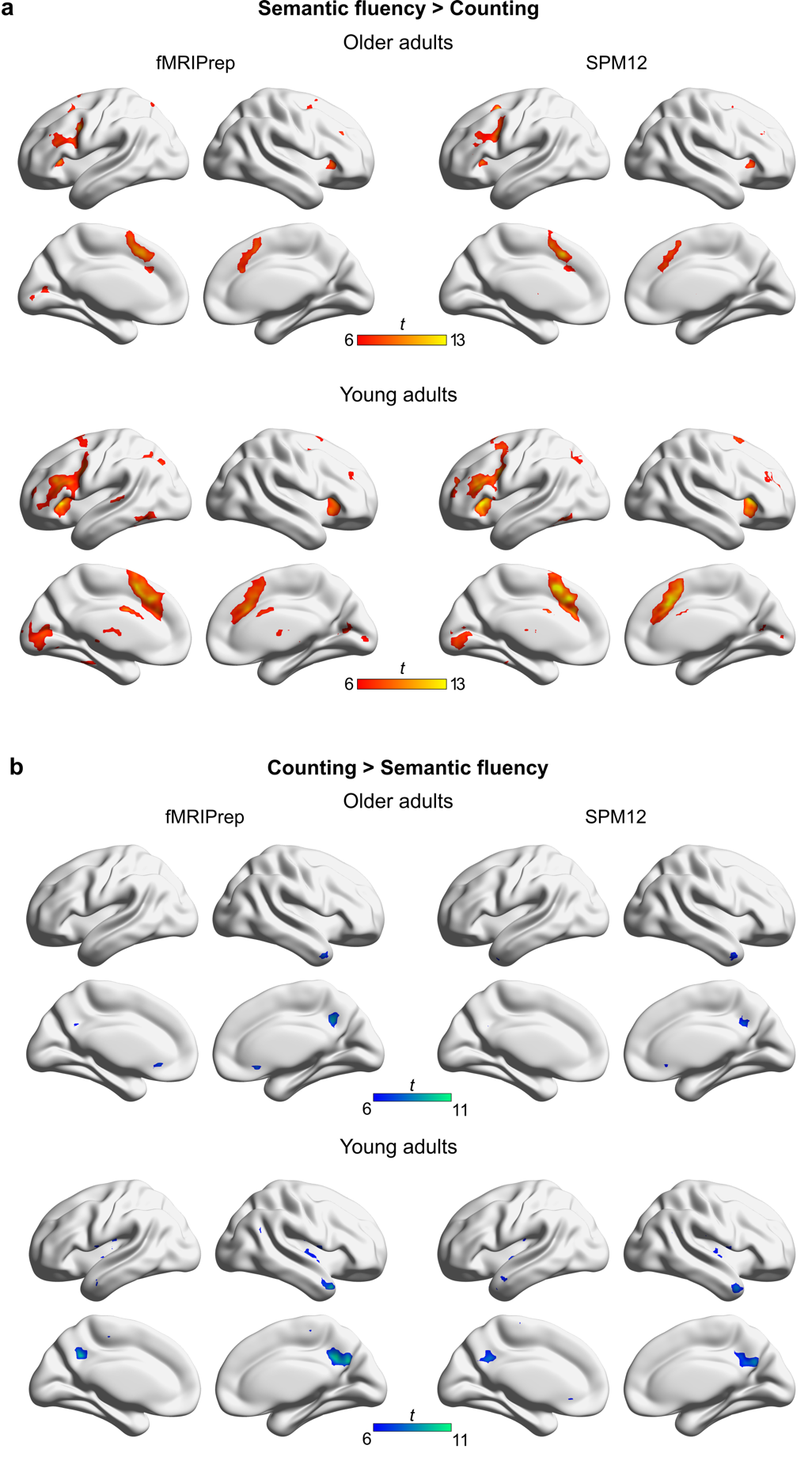


**Figure S1. Comparison of different preprocessing pipelines.** Plots show univariate results for preprocessing with fMRIPrep 20.2.3 and SPM12 in each age group for contrasts (A) Semantic fluency > Counting and (B) Counting > Semantic fluency. Results are FWE-corr. p < 0.05 at peak-level with a minimum cluster size of k = 10 voxels.

##
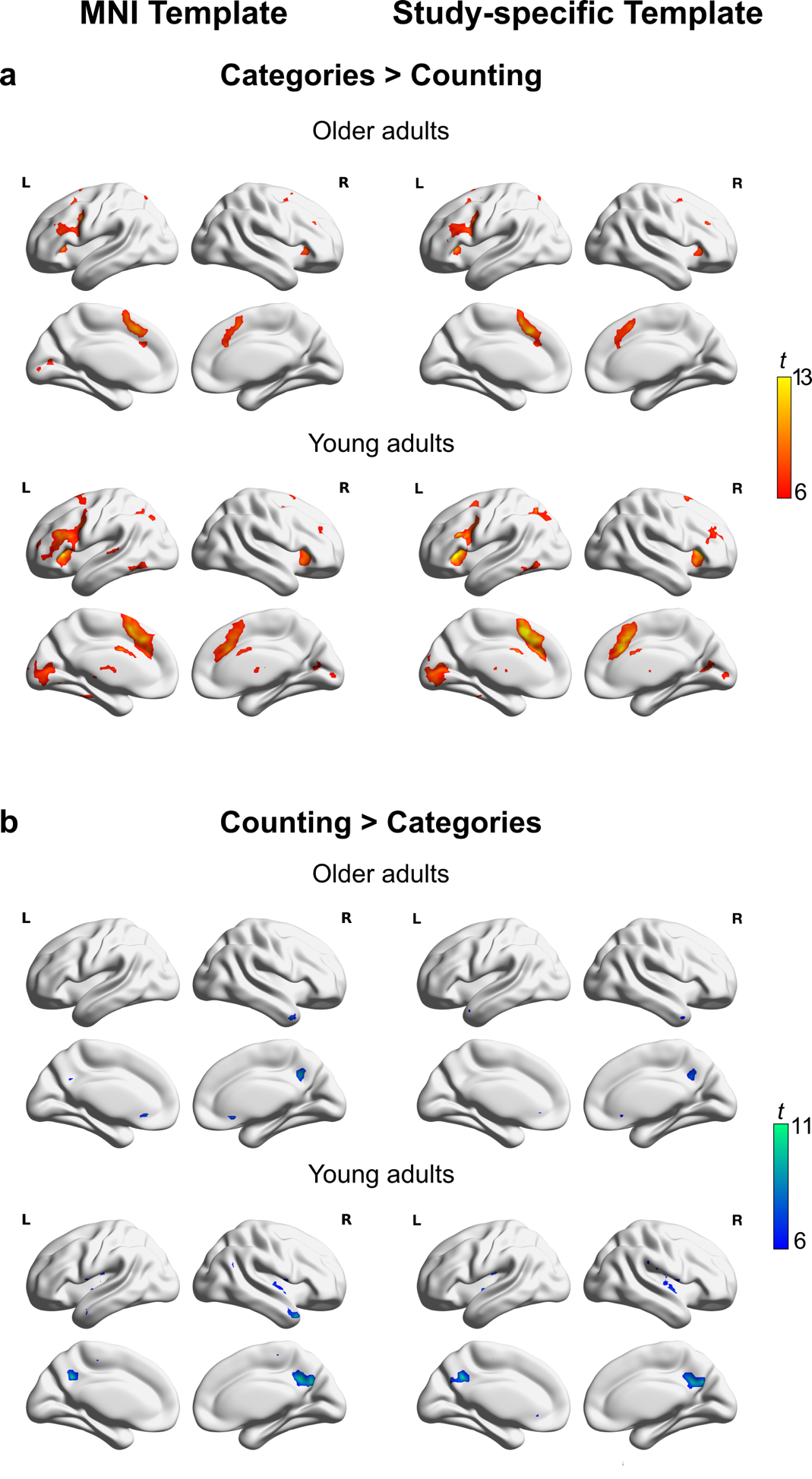
Figure S2

**Figure S 2. Comparison of within-group task effects for two different resampling procedures:** Using the MNI standard template and a study-specific template. Results are FWE-corr. p < 0.05 at peak-level with a minimum cluster size of k = 10 voxels.

## Figure S3


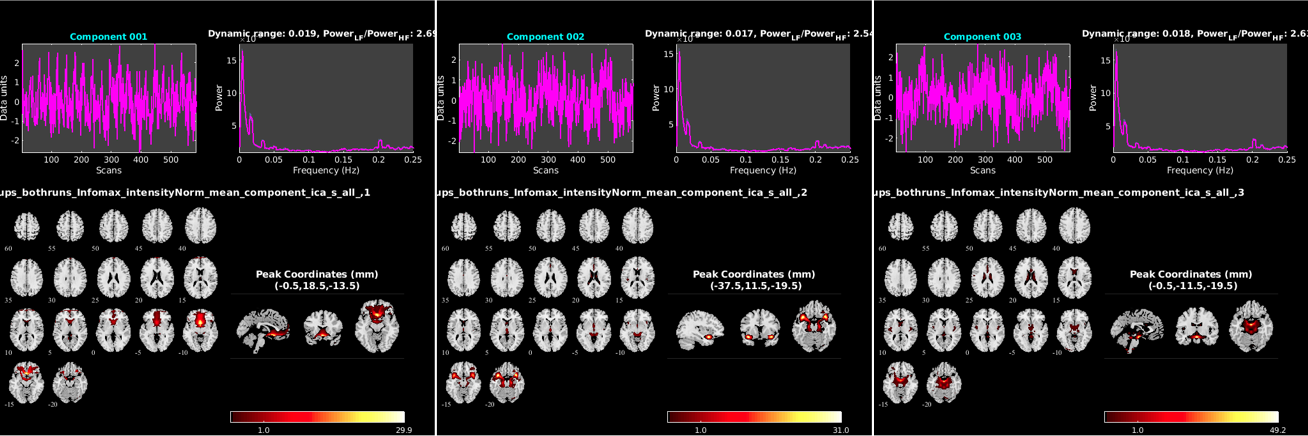


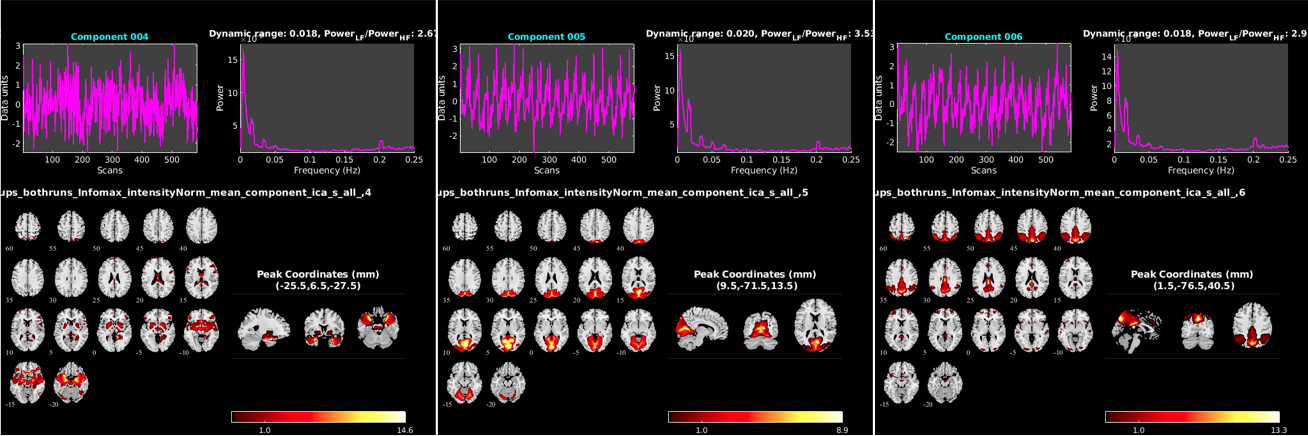


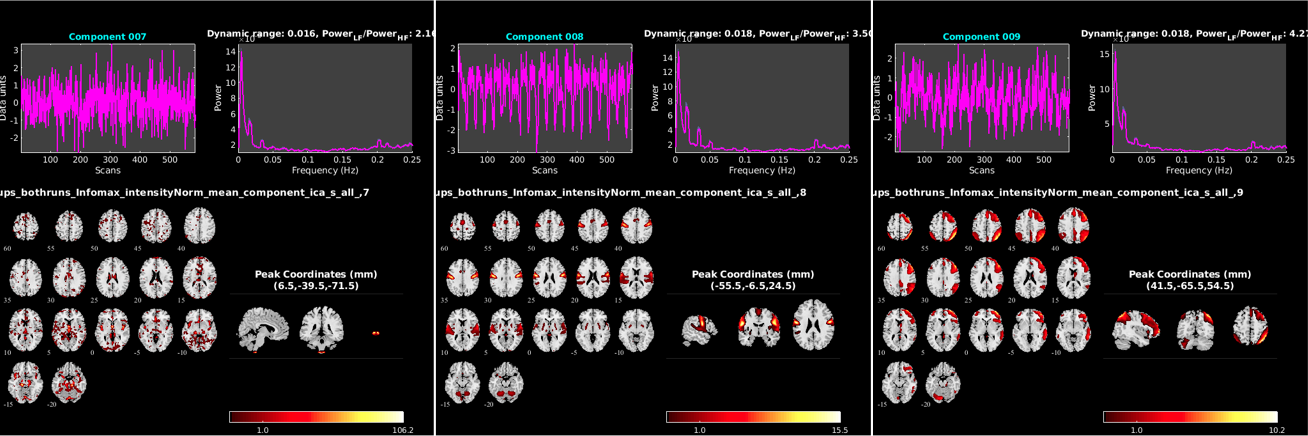


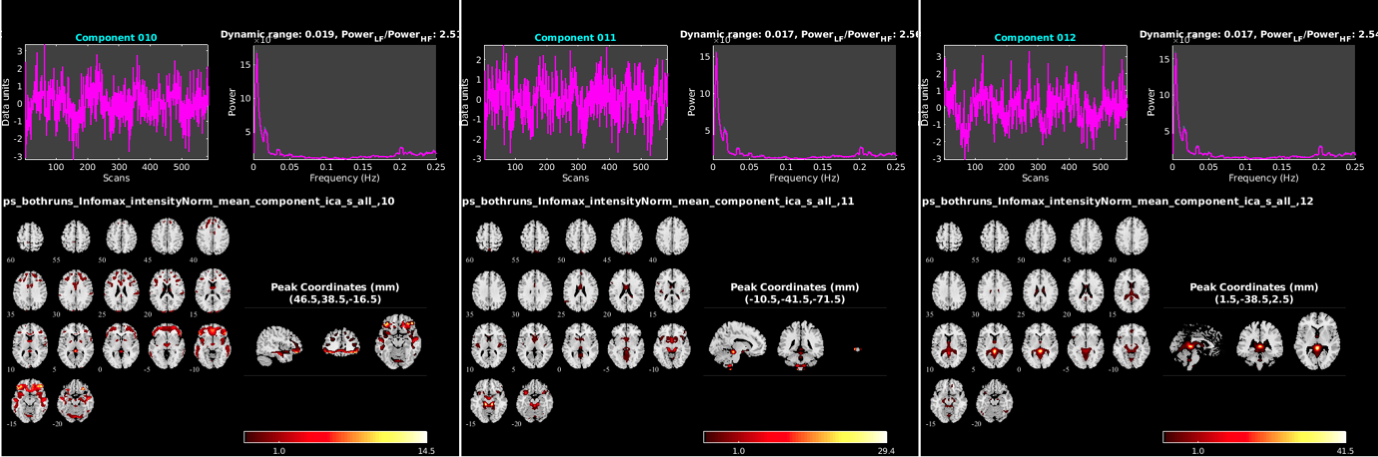


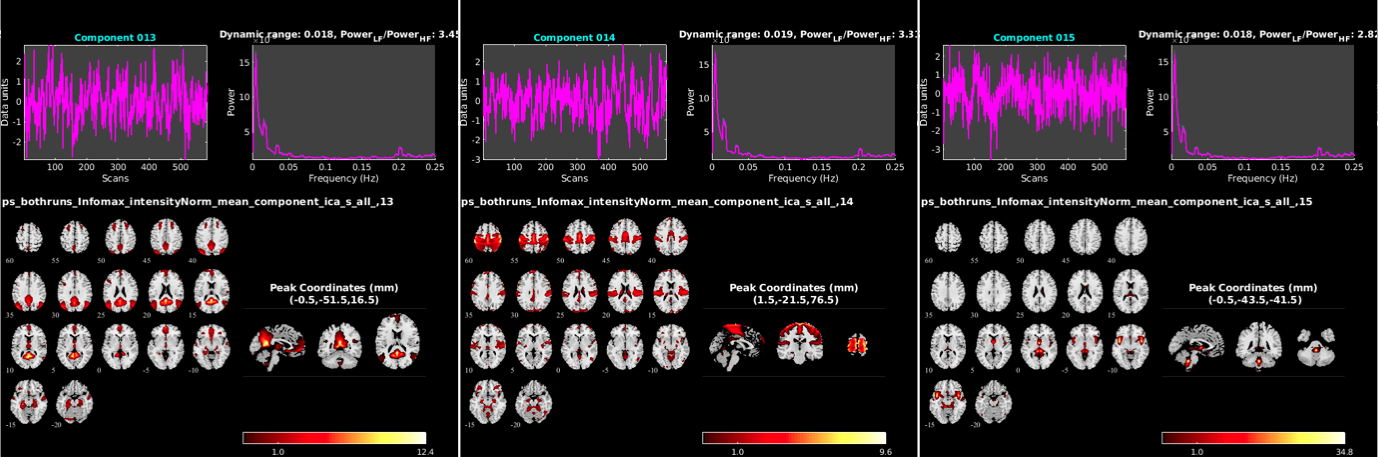


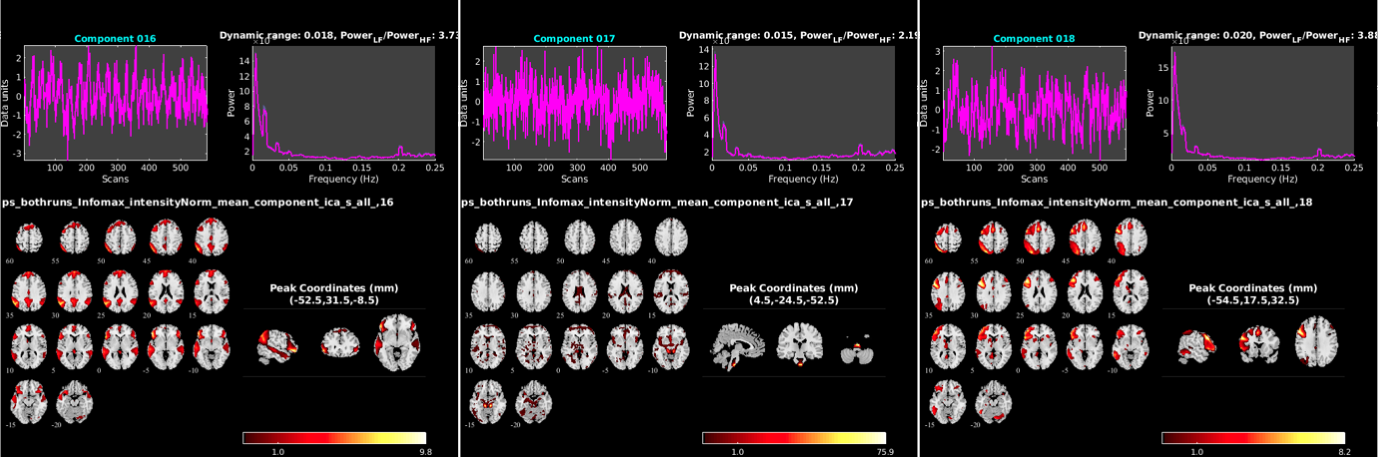


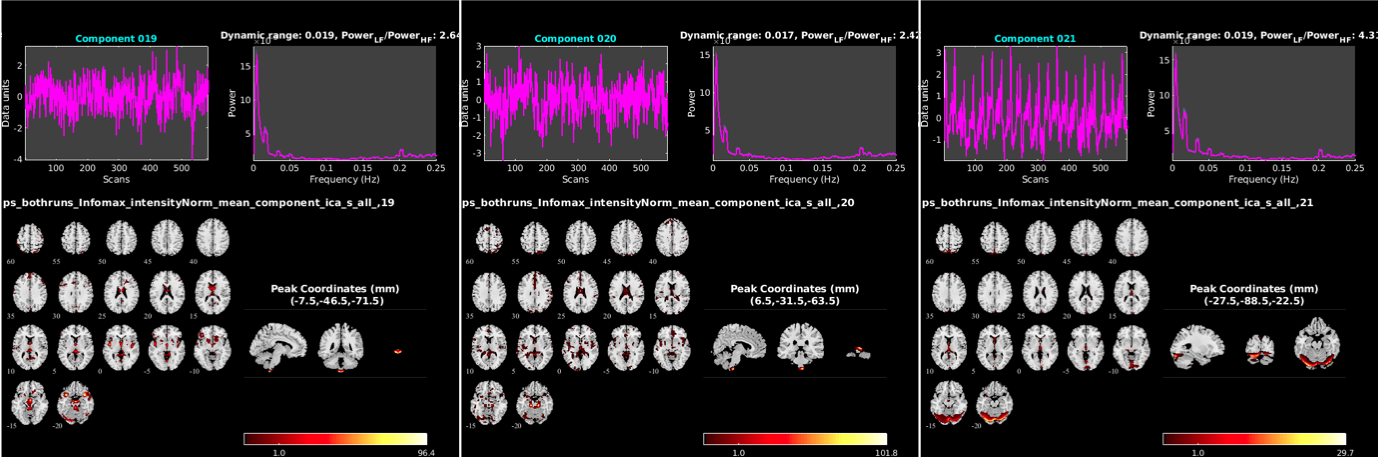


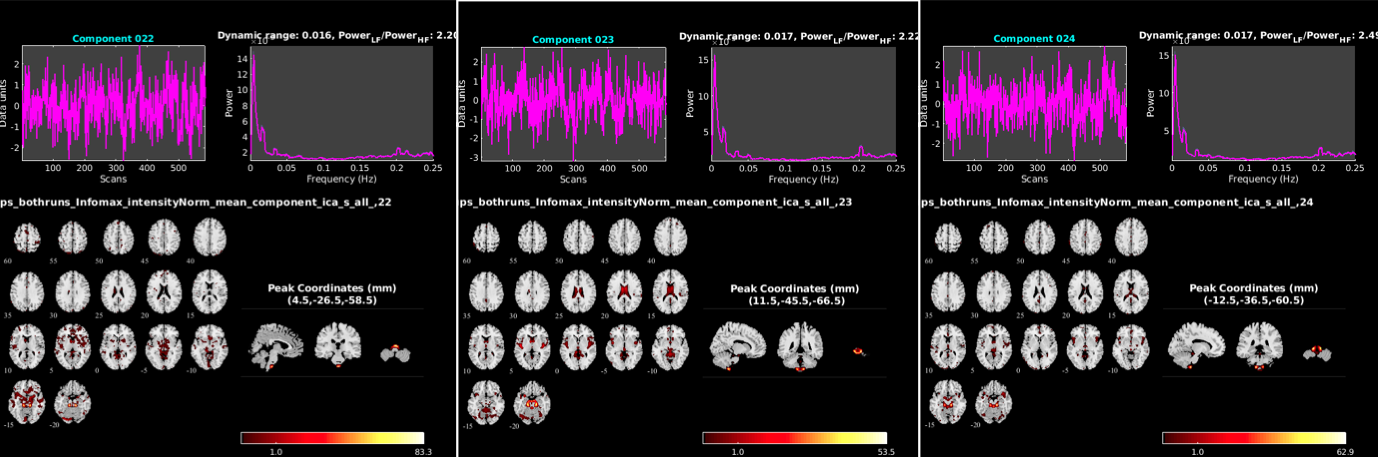


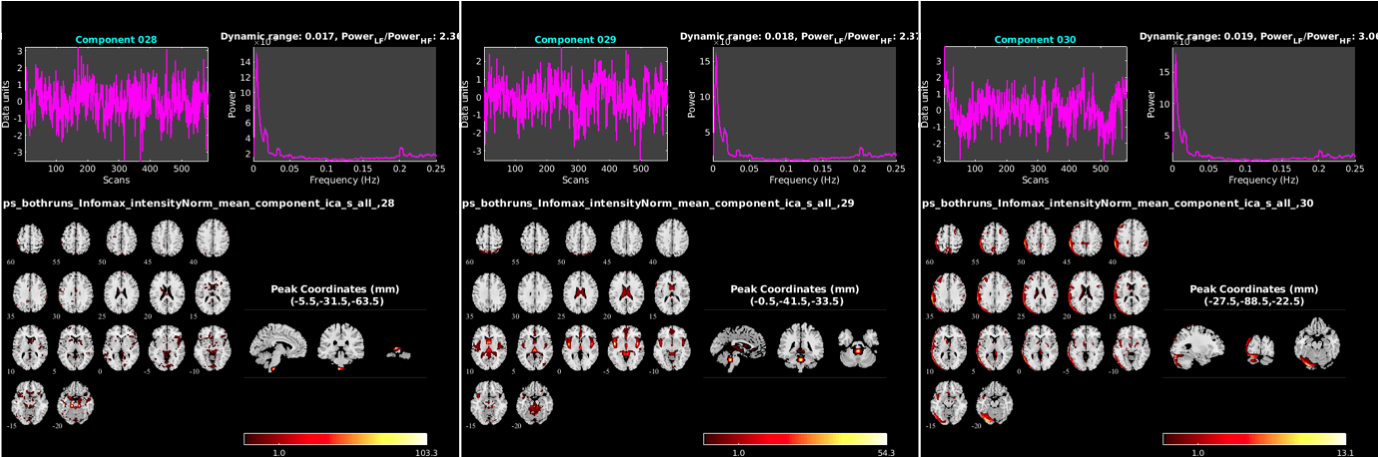


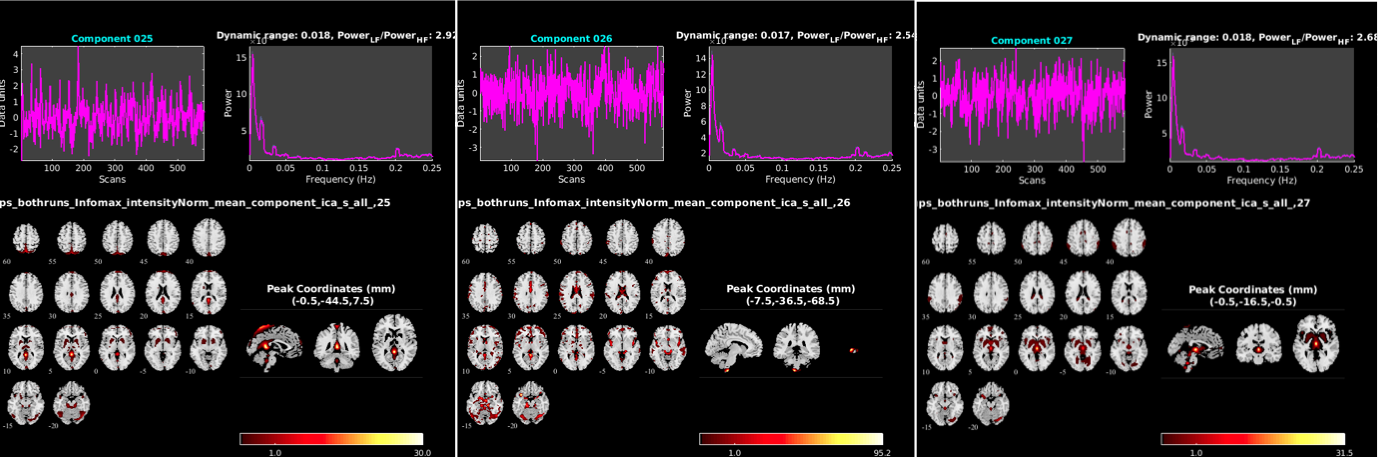


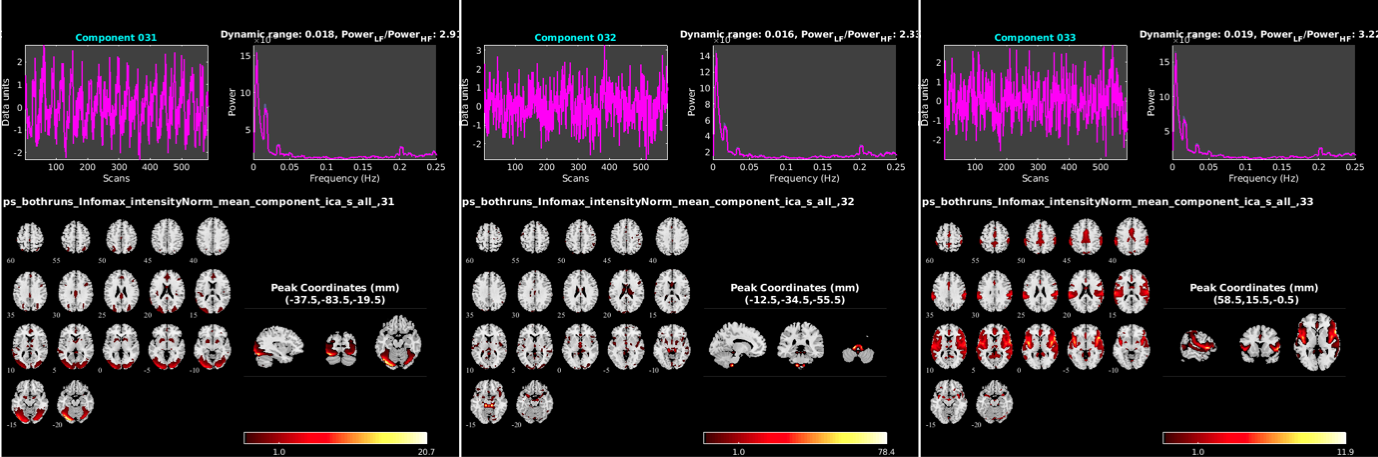


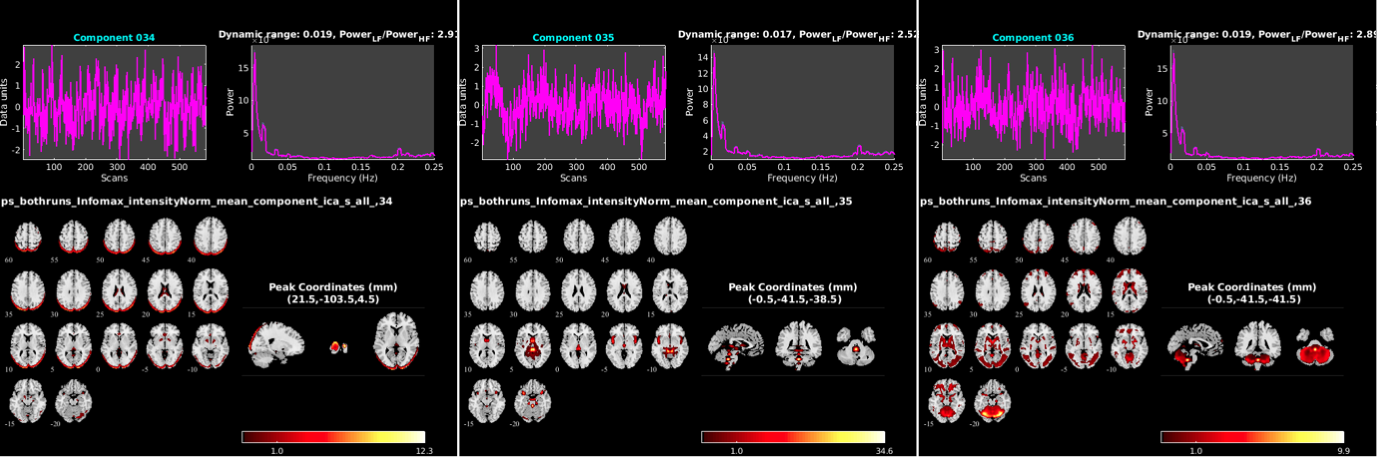


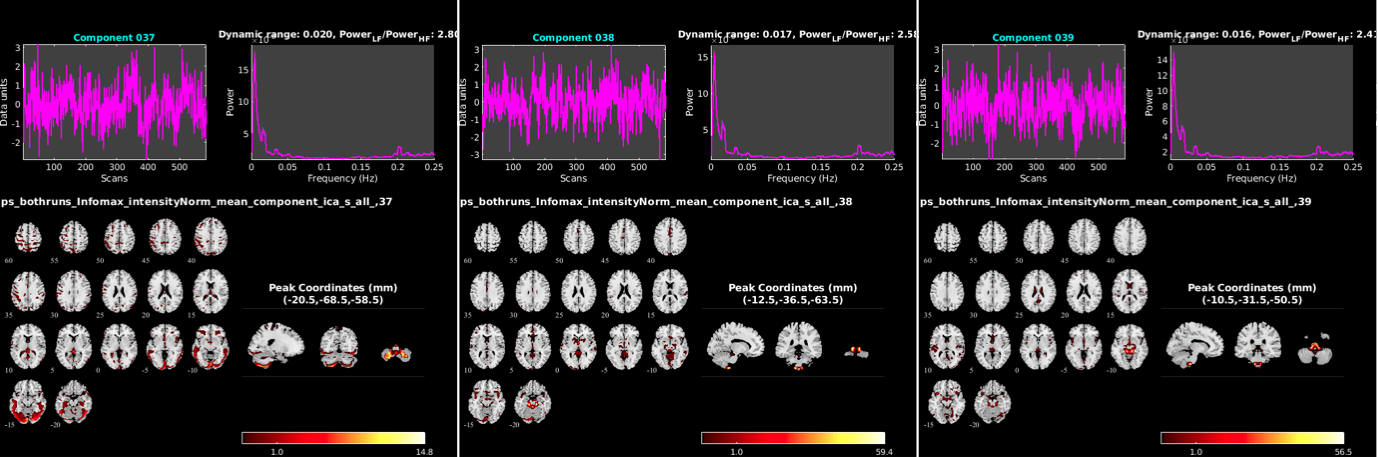


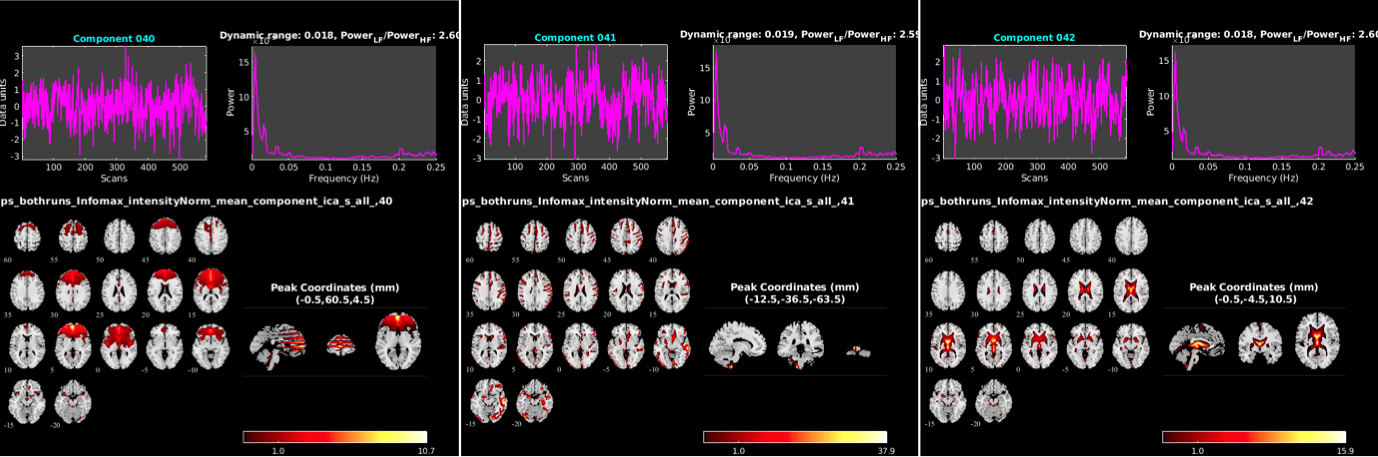


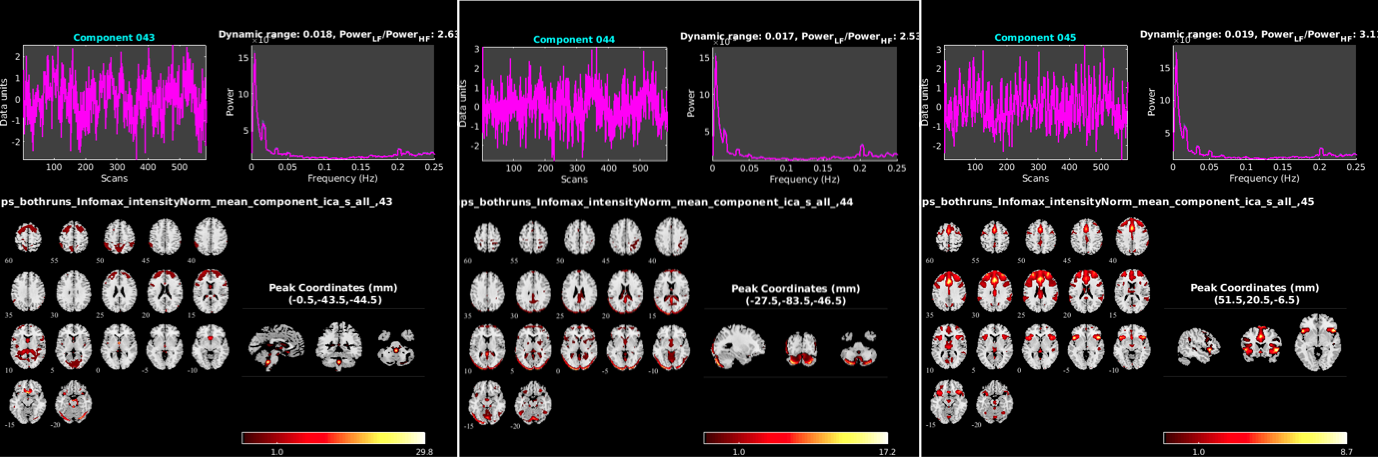


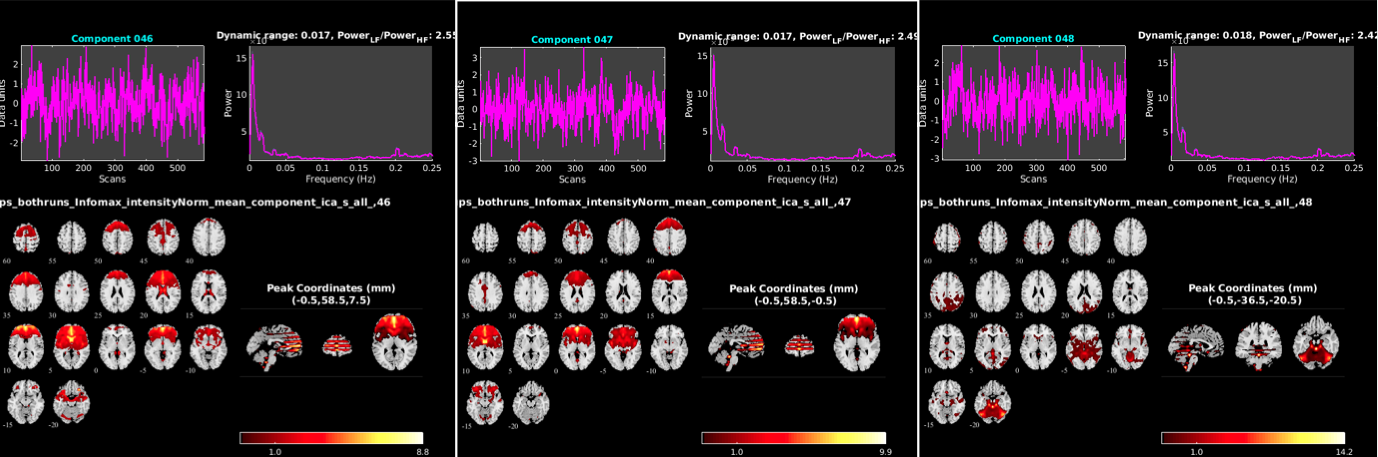


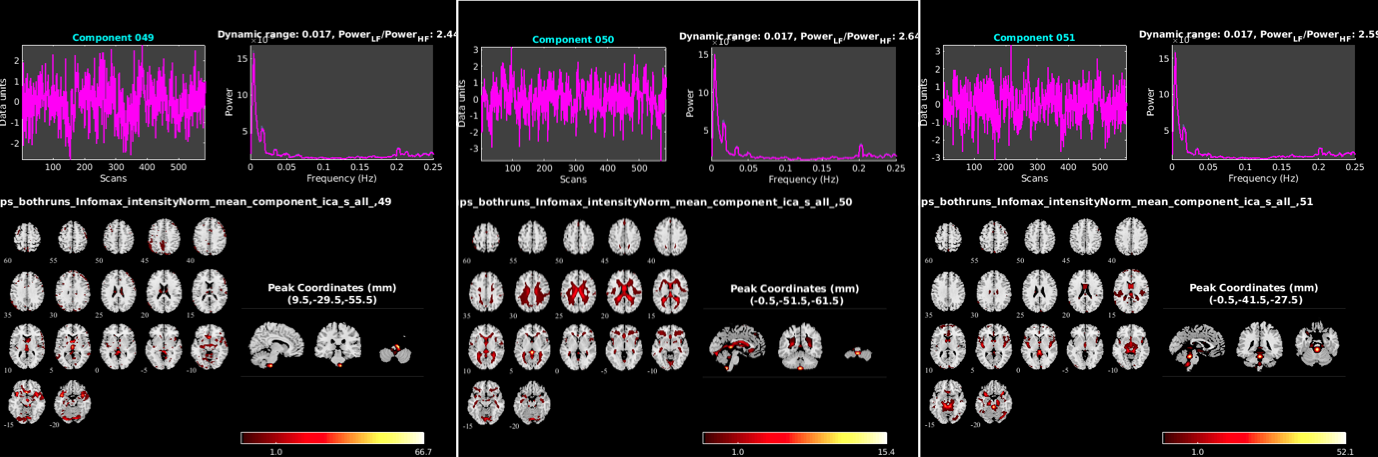


**
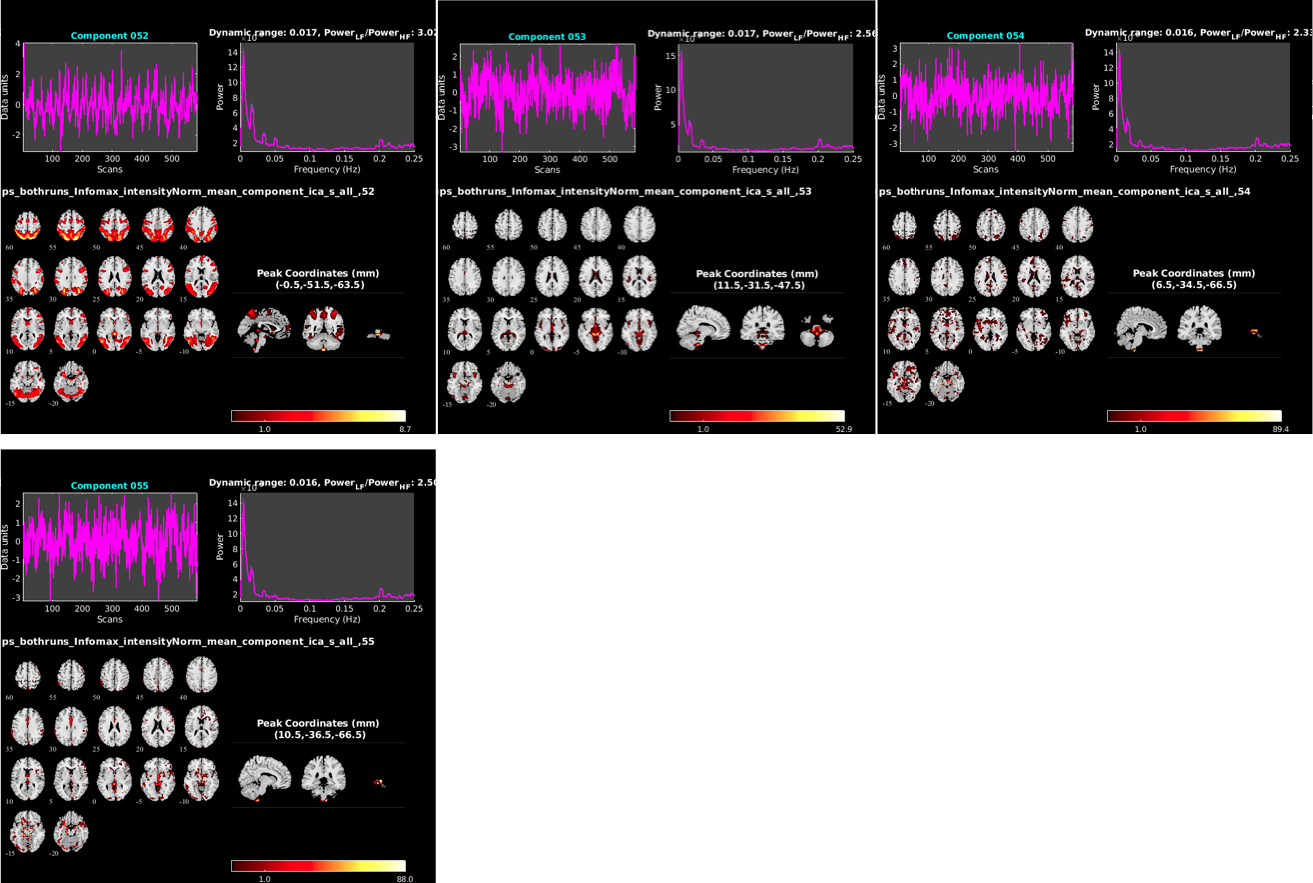
Figure S3. 55 independent network components derived via group spatial independent component analysis (ICA).** ICA was conducted using the Group ICA of fMRI Toolbox. Dimensions were reduced to 55 using minimum description length information criteria. Icasso was repeated 50 times to ensure reliability of the decomposition, and group-level ICs were back-reconstructed to the participant level using the group-information guided ICA (GICA3) algorithm.

## Figure S4

**Figure S4. Plots of global cost efficiency (GCE) against cost for different filtering options of graphs.** We compared the GCE of two topological (minimum spanning tree (MST) and orthogonal minimum spanning tree (OMST)) and one proportional (5-20% strongest edge weights) edge filtering method in both age groups. Symbols indicate the average value in each group while densities display value distribution.

**
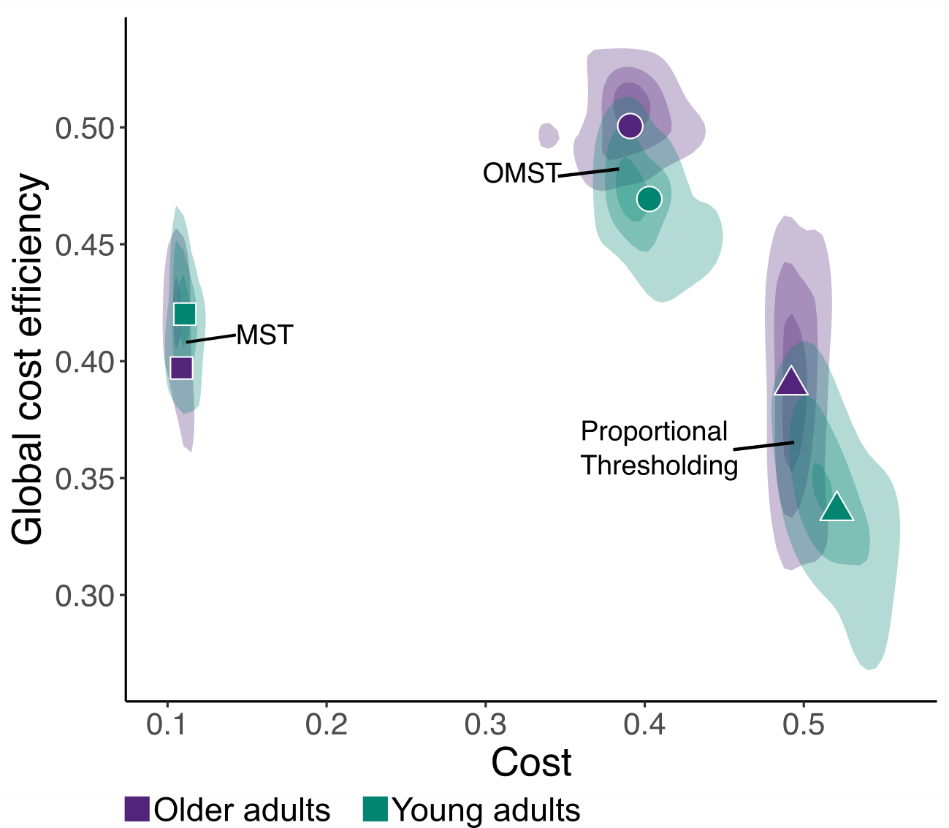
**

## Figure S5

**
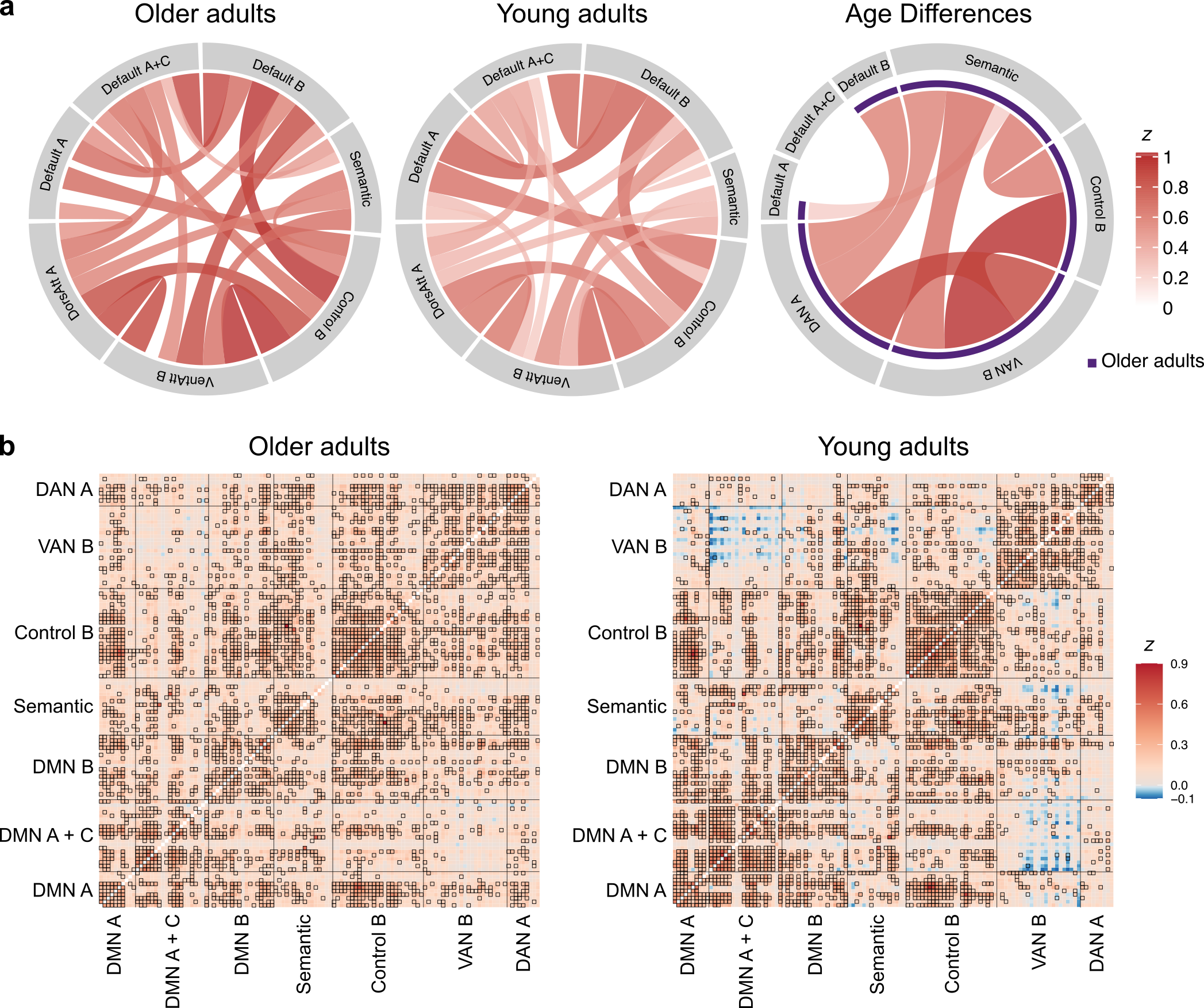
Figure S5. Within- and between-network functional connectivity results for denoising pipeline without global signal regression.** (a) Chord diagrams display significant results of functional coupling for whole ICA-derived networks. Age differences were assessed using permutation testing in network-based statistics (cluster-forming threshold at p = 0.01, FWE-corrected significance threshold at p = 0.025 with 10,000 permutations). (b) Heatmaps show functional coupling between individual regions of interest (n = 121) of the seven networks. Note that there were no significant age differences at p = 0.025.

## Figure S6

**
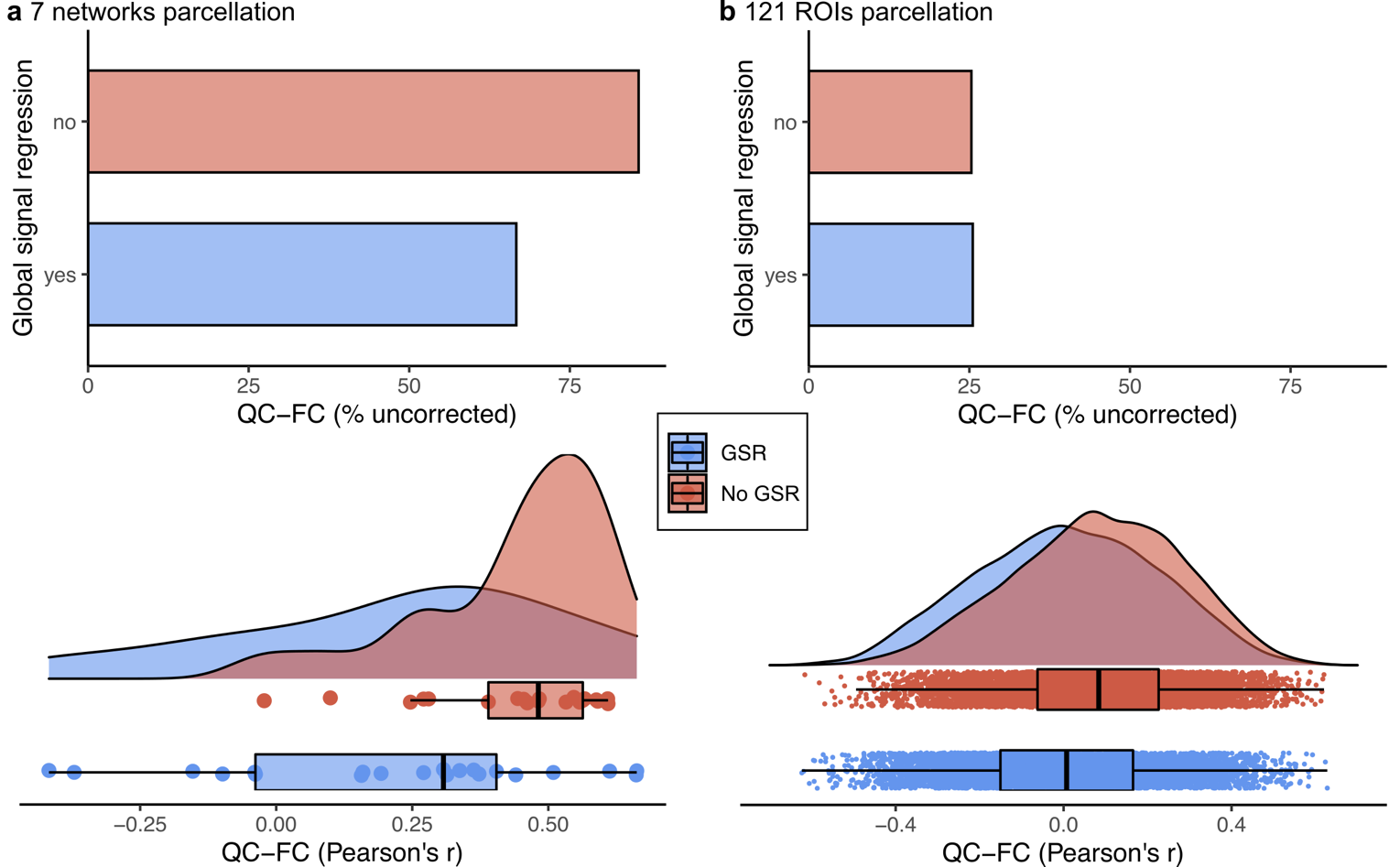
**

**Figure S6. Comparison of denoising pipelines with global signal regression (GSR) and without for association between motion (mean FD) and functional connectivity (FC).** The upper panels display the proportion of FC values that are correlated to mean FD at a threshold p < 0.05, uncorrected. The bottom panels show the distribution of all QC-FC correlations as smoothed kernel density estimates and boxplots with medians and interquartile ranges.

# Supplementary Results

## Figure S7

**
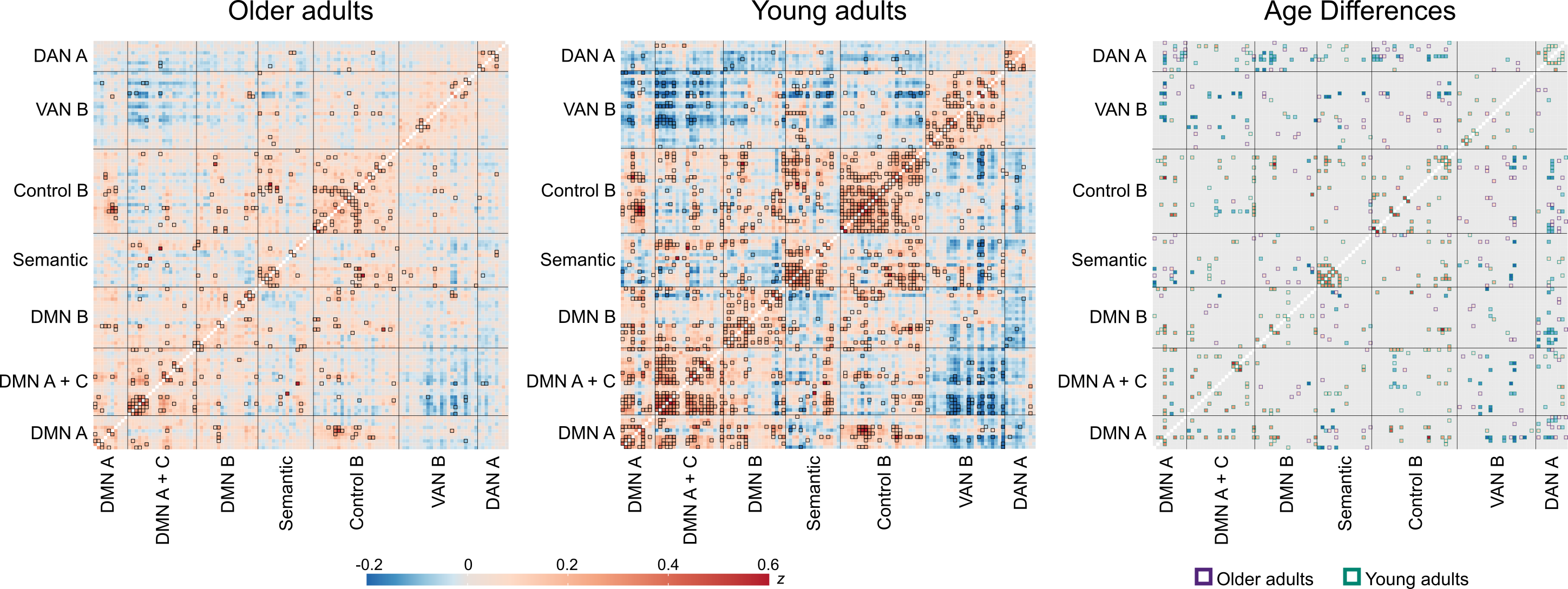
**

**Figure S7. Heatmaps display significant results of functional coupling between the regions of interest (n = 121) of the ICA-derived networks.** Connectivity values are Fisher-transformed partial correlations. Bold frames indicate significant values which are based on cPPI-derived significance values in the age groups while age differences were assessed using permutation testing in network-based statistics (cluster-forming threshold at p = 0.01, FWE-corrected significance threshold at p = 0.025 with 10,000 permutations).

Table S1**.** Behavioral results of mixed-effects models for accuracy and response time

|  | **Accuracy** | | | | **Response time** | | | |
| --- | --- | --- | --- | --- | --- | --- | --- | --- |
| *Coefficient* | *Log-Odds* | *CI* | *z-value* | *p* | *Estimates* | *CI* | *t-value* | *p* |
| Intercept | 4.63 | 4.15 – 5.10 | 19.09 | **<0.001** | 6.43 | 6.39 – 6.47 | 326.96 | **<0.001** |
| Age | -0.68 | -1.13 – -0.23 | -2.98 | **0.003** | 0.01 | 0.00 – 0.03 | 2.30 | **0.021** |
| Condition | -3.16 | -4.06 – -2.26 | -6.90 | **<0.001** | 0.15 | 0.10 – 0.19 | 6.29 | **<0.001** |
| Difficulty | 2.01 | 1.50 – 2.51 | 7.76 | **<0.001** | -0.07 | -0.10 – -0.04 | -4.71 | **<0.001** |
| Education | -0.11 | -0.24 – 0.01 | -1.75 | 0.080 | -0.01 | -0.01 – 0.00 | -1.88 | 0.060 |
| Age * Condition | 0.64 | -0.18 – 1.46 | 1.53 | 0.125 | 0.08 | 0.06 – 0.10 | 8.94 | **<0.001** |
| Age * Difficulty | -0.67 | -1.08 – -0.26 | -3.19 | **0.001** | -0.00 | -0.02 – 0.01 | -0.39 | 0.695 |
| **Random Effects** | | | | | | | | |
| σ^2^ | 3.29 | | | | 0.09 | | | |
| τ_00_ | 0.19 _Subj_ | | | | 0.01 _Subj_ | | | |
|  | 0.29 _Category_ | | | | 0.00 _Category_ | | | |
| ICC | 0.13 | | | | 0.09 | | | |
| Observations | 19710 | | | | 18877 | | | |
| Marginal R^2^ / Conditional R^2^ | 0.495 / 0.559 | | | | 0.065 / 0.148 | | | |

*Note*. Significant effects are marked in bold. Contrasts are sum coded. P-values were obtained via likelihood ratio tests. CI Confidence interval.

Table S2. Jaccard similarity coefficients with the 7-networks parcellation scheme

|  | **IC06** | **IC09** | **IC13** | **IC16** | **IC18** | **IC45** | **IC52** |
| --- | --- | --- | --- | --- | --- | --- | --- |
| Control | 0.128 | **0.287** | 0.039 | 0.054 | *0.194* | *0.161* | 0.090 |
| Default | **0.155** | *0.154* | **0.234** | **0.412** | 0.133 | 0.133 | 0.043 |
| Dorsal attention | 0.073 | 0.089 | 0.067 | 0.022 | 0.139 | 0.059 | **0.301** |
| Limbic | 0.000 | 0.004 | 0.012 | 0.014 | 0.008 | 0.005 | 0.011 |
| Ventral attention | 0.039 | 0.045 | 0.025 | 0.039 | 0.053 | **0.211** | 0.097 |
| Somatomotor | 0.017 | 0.064 | 0.029 | 0.057 | 0.067 | 0.044 | 0.052 |
| Visual | 0.038 | 0.015 | 0.090 | 0.046 | 0.045 | 0.028 | *0.177* |
| General semantic cognition | 0.032 | 0.030 | 0.072 | *0.194* | **0.201** | 0.092 | 0.050 |
| Semantic control | 0.012 | 0.036 | 0.012 | 0.067 | *0.153* | 0.091 | 0.027 |

*Note.* The selected network labels for the respective independent components are shown in bold while all cognitive networks that showed a higher similarity coefficient than *J* = 0.15 are shown in italics.

Table S3. Significant clusters of task-relevant independent components

| **Anatomical structure** | **Hemi** | ***k*** | ***t*** | ***x*** | ***y*** | ***z*** |
| --- | --- | --- | --- | --- | --- | --- |
| **IC 06** |  |  |  |  |  |  |
| **Precuneus Cortex** | **R** | **1057** | **19.94** | **5** | **-71** | **35** |
| Precuneus Cortex | L |  | 17.67 | -5 | -66 | 38 |
| Cingulate Gyrus, posterior division | R |  | 17.43 | 2 | -41 | 35 |
| **Angular Gyrus** | **L** | **242** | **14.56** | **-45** | **-64** | **46** |
| Angular Gyrus | L |  | 13.29 | -48 | -56 | 44 |
| **Angular Gyrus** | **R** | **289** | **14.31** | **47** | **-56** | **44** |
| Angular Gyrus | R |  | 10.44 | 54 | -51 | 41 |
| Angular Gyrus | R |  | 9.58 | 52 | -54 | 33 |
| **Cingulate Gyrus, anterior division** | **R** | **51** | **7.58** | **2** | **43** | **5** |
| Paracingulate Gyrus | R |  | 6.17 | 5 | 46 | -3 |
| **Paracingulate Gyrus** | **L** | **25** | **6.96** | **-3** | **38** | **22** |
| **Frontal Pole** | **L** | **16** | **6.43** | **-23** | **66** | **5** |
| **IC 09** |  |  |  |  |  |  |
| **Frontal Pole** | **R** | **564** | **21.83** | **42** | **48** | **-9** |
| Frontal Pole | R |  | 20.52 | 49 | 43 | -9 |
| Frontal Pole | R |  | 19.22 | 29 | 56 | 5 |
| **Middle Frontal Gyrus** | **R** | **810** | **21.33** | **49** | **31** | **33** |
| Middle Frontal Gyrus | R |  | 18.52 | 37 | 21 | 55 |
| Middle Frontal Gyrus | R |  | 18.25 | 52 | 16 | 41 |
| **Angular Gyrus** | **R** | **520** | **20.75** | **52** | **-51** | **44** |
| Angular Gyrus | R |  | 20.72 | 57 | -56 | 38 |
| Angular Gyrus | R |  | 20.26 | 39 | -61 | 52 |
| **Middle Temporal Gyrus, posterior division** | **R** | **104** | **18.62** | **62** | **-36** | **-12** |
| Middle Temporal Gyrus, temporooccipital part | R |  | 16.94 | 59 | -46 | -12 |
| Middle Temporal Gyrus, temporooccipital part | R |  | 14.67 | 67 | -39 | -3 |
| **Superior Frontal Gyrus** | **R** | **335** | **18.38** | **5** | **36** | **44** |
| Paracingulate Gyrus | R |  | 18.04 | 7 | 26 | 46 |
| Superior Frontal Gyrus | L |  | 10.93 | -5 | 33 | 46 |
| **Angular Gyrus** | **L** | **364** | **15.31** | **-48** | **-54** | **55** |
| Angular Gyrus | L |  | 12.43 | -45 | -61 | 52 |
| Angular Gyrus | L |  | 11.15 | -38 | -59 | 46 |
| **Frontal Pole** | **L** | **231** | **14.18** | **-43** | **53** | **2** |
| Frontal Pole | L |  | 13.29 | -38 | 61 | -1 |
| Frontal Pole | L |  | 10.39 | -48 | 46 | -12 |
| **Middle Frontal Gyrus** | **L** | **200** | **10.80** | **-50** | **26** | **35** |
| Middle Frontal Gyrus | L |  | 10.27 | -48 | 21 | 44 |
| **Middle Temporal Gyrus, posterior division** | **L** | **41** | **7.73** | **-63** | **-36** | **-9** |
| **Superior Frontal Gyrus** | **L** | **10** | **6.08** | **-23** | **18** | **57** |
| **IC 13** |  |  |  |  |  |  |
| **Precuneus Cortex** | **L** | **1459** | **32.20** | **-5** | **-59** | **19** |
| Precuneus Cortex | R |  | 32.11 | 5 | -59 | 27 |
| Precuneus Cortex | R |  | 26.92 | 7 | -54 | 16 |
| **Angular Gyrus** | **L** | **525** | **20.64** | **-48** | **-71** | **30** |
| Angular Gyrus | L |  | 18.65 | -40 | -66 | 27 |
| Angular Gyrus | L |  | 14.87 | -38 | -79 | 41 |
| **Parahippocampal Gyrus, posterior division** | **L** | **207** | **18.74** | **-25** | **-31** | **-17** |
| Parahippocampal Gyrus, anterior division | L |  | 10.87 | -20 | -22 | -20 |
| Lingual Gyrus | L |  | 10.86 | -25 | -41 | -6 |
| **Superior Frontal Gyrus** | **L** | **228** | **16.63** | **-23** | **28** | **49** |
| **Angular Gyrus** | **R** | **461** | **15.89** | **52** | **-64** | **24** |
| Angular Gyrus | R |  | 13.36 | 54 | -69 | 35 |
| Angular Gyrus | R |  | 11.74 | 44 | -54 | 22 |
| **Frontal Medial Cortex** | **L** | **718** | **15.37** | **-5** | **51** | **-9** |
| Cingulate Gyrus, anterior division | R |  | 13.77 | 2 | 33 | -9 |
| Frontal Medial Cortex | R |  | 12.56 | 2 | 53 | -6 |
| **Middle Temporal Gyrus, anterior division** | **R** | **109** | **14.58** | **64** | **1** | **-17** |
| **Parahippocampal Gyrus, posterior division** | **R** | **133** | **11.99** | **27** | **-31** | **-17** |
| Parahippocampal Gyrus, anterior division | R |  | 11.68 | 22 | **-19** | -23 |
| **Superior Frontal Gyrus** | **R** | **185** | **11.08** | **22** | **31** | **46** |
| **IC 16** |  |  |  |  |  |  |
| **Frontal Pole** | **L** | **1390** | **23.11** | **-5** | **48** | **46** |
| Superior Frontal Gyrus | R |  | 21.03 | 14 | 31 | 57 |
| Paracingulate Gyrus | L |  | 20.26 | -5 | 53 | 19 |
| **Middle Temporal Gyrus, anterior division** | **L** | **1382** | **23.08** | **-55** | **1** | **-20** |
| Middle Temporal Gyrus, posterior division | L |  | 21.78 | -58 | -31 | -3 |
| Frontal Pole | L |  | 19.89 | -50 | 43 | -12 |
| **Angular Gyrus** | **L** | **356** | **22.67** | **-55** | **-59** | **30** |
| Supramarginal Gyrus, posterior division | L |  | 14.15 | -63 | -49 | 41 |
| **Inferior Frontal Gyrus, pars triangularis** | **R** | **313** | **17.26** | **52** | **31** | **-6** |
| Temporal Pole | R |  | 12.61 | 37 | 23 | -23 |
| Frontal Orbital Cortex | R |  | 11.27 | 44 | 23 | -14 |
| **Temporal Pole** | **R** | **238** | **16.84** | **52** | **16** | **-25** |
| Middle Temporal Gyrus, anterior division | R |  | 16.64 | 52 | 3 | -31 |
| Temporal Pole | R |  | 15.86 | 49 | 11 | -34 |
| **Middle Temporal Gyrus, posterior division** | **R** | **193** | **16.70** | **64** | **-29** | **-3** |
| Middle Temporal Gyrus, posterior division | R |  | 15.08 | 54 | -24 | -6 |
| Middle Temporal Gyrus, posterior division | R |  | 14.76 | 67 | -36 | -1 |
| **Middle Frontal Gyrus** | **L** | **151** | **16.01** | **-40** | **16** | **52** |
| **IC 18** |  |  |  |  |  |  |
| **Insular Cortex** | **L** | **1554** | **28.83** | **-33** | **21** | **-1** |
| Inferior Frontal Gyrus, pars triangularis | L |  | 21.00 | -48 | 28 | 19 |
| Middle Frontal Gyrus | L |  | 19.65 | -50 | 21 | 33 |
| **Superior Frontal Gyrus** | **L** | **511** | **22.76** | **-5** | **31** | **44** |
| Paracingulate Gyrus | L |  | 19.99 | -5 | 13 | 52 |
| Paracingulate Gyrus | R |  | 18.29 | 2 | 21 | 46 |
| **Inferior Temporal Gyrus, temporooccipital part** | **L** | **984** | **18.99** | **-58** | **-49** | **-12** |
| Superior Temporal Gyrus, posterior division | L |  | 10.54 | -60 | -31 | 5 |
| Parahippocampal Gyrus, posterior division | L |  | 9.82 | -30 | -31 | -17 |
| **Angular Gyrus** | **L** | **270** | **16.99** | **-35** | **-59** | **41** |
| Angular Gyrus | L |  | 16.88 | -30 | -66 | 49 |
| Angular Gyrus | L |  | 16.52 | -30 | -71 | 41 |
| **Superior Frontal Gyrus** | **L** | **50** | **11.92** | **-23** | **26** | **46** |
| Superior Frontal Gyrus | L |  | 9.63 | -15 | 36 | 46 |
| Frontal Pole | L |  | 8.39 | -13 | 48 | 44 |
| **Temporal Fusiform Cortex, anterior division** | **L** | **22** | **9.50** | **-38** | **-9** | **-28** |
| **Superior Temporal Gyrus, anterior division** | **R** | **32** | **9.14** | **59** | **-4** | **-1** |
| **IC 45** |  |  |  |  |  |  |
| **Frontal Pole** | **L** | **583** | **23.44** | **-25** | **38** | **30** |
| Frontal Pole | L |  | 21.05 | -23 | 46 | 24 |
| Frontal Pole | L |  | 19.36 | -30 | 53 | 24 |
| **Paracingulate Gyrus** | **L** | **565** | **21.83** | **-5** | **31** | **33** |
| Cingulate Gyrus, anterior division | L |  | 21.81 | -5 | 31 | 22 |
| Paracingulate Gyrus | R |  | 21.76 | 14 | 28 | 27 |
| **Frontal Pole** | **R** | **630** | **20.80** | **27** | **48** | **30** |
| Frontal Pole | R |  | 20.35 | 27 | 41 | 24 |
| Frontal Pole | R |  | 18.56 | 37 | 43 | 30 |
| **Frontal Operculum Cortex** | **L** | **225** | **16.57** | **-35** | **16** | **11** |
| Frontal Orbital Cortex | L |  | 15.46 | -33 | 26 | -6 |
| Inferior Frontal Gyrus, pars opercularis | L |  | 15.04 | -50 | 13 | -3 |
| **Frontal Operculum Cortex** | **R** | **280** | **16.15** | **47** | **18** | **-3** |
| Frontal Operculum Cortex | R |  | 15.26 | 39 | 18 | 11 |
| Frontal Orbital Cortex | R |  | 14.28 | 39 | 18 | -12 |
| **Supramarginal Gyrus, posterior division** | **R** | **157** | **14.98** | **62** | **-44** | **27** |
| Supramarginal Gyrus, posterior division | R |  | 13.20 | 67 | -39 | 35 |
| Supramarginal Gyrus, posterior division | R |  | 5.93 | 62 | -39 | 49 |
| **Superior Frontal Gyrus** | **L** | **27** | **14.57** | **-13** | **3** | **68** |
| Superior Frontal Gyrus | L |  | 12.61 | -8 | 8 | 63 |
| **Supramarginal Gyrus, posterior division** | **L** | **41** | **11.77** | **-60** | **-44** | **27** |
| **Inferior Frontal Gyrus, pars opercularis** | **R** | **22** | **8.01** | **52** | **11** | **11** |
| Inferior Frontal Gyrus, pars opercularis | R |  | 6.73 | 54 | 13 | 22 |
| **Frontal Pole** | **L** | **27** | **7.76** | **-30** | **46** | **-14** |
| **Inferior Frontal Gyrus, pars opercularis** | **L** | **17** | **7.00** | **-50** | **11** | **11** |
| **IC 52** |  |  |  |  |  |  |
| **Angular Gyrus** | **R** | **1860** | **22.56** | **27** | **-69** | **44** |
| Angular Gyrus | R |  | 21.55 | 39 | -76 | 27 |
| Inferior Temporal Gyrus, temporooccipital part | R |  | 20.54 | 52 | -59 | -12 |
| **Lateral Occipital Cortex, inferior division** | **L** | **1437** | **21.63** | **-48** | **-66** | **2** |
| Angular Gyrus | L |  | 21.17 | -43 | -81 | 16 |
| Angular Gyrus | L |  | 20.11 | -28 | -76 | 30 |
| **Temporal Occipital Fusiform Cortex** | **L** | **24** | **12.07** | **-28** | **-51** | **-12** |
| Temporal Fusiform Cortex, posterior division | L |  | 8.92 | -30 | -41 | -12 |
| **Temporal Occipital Fusiform Cortex** | **R** | **15** | **8.49** | **37** | **-44** | **-23** |

*Note.* Results are based on one-sided t-tests and FWE-corrected p < 0.05 at peak level with a cluster extent threshold k = 10.

Table S4. Results for significant effects of cPPI connectivity for accuracy

|  | **Accuracy** | | | | **Accuracy** | | | |
| --- | --- | --- | --- | --- | --- | --- | --- | --- |
| *Coefficient* | *Log-Odds* | *CI* | *Statistic* | *p* | *Log-Odds* | *CI* | *Statistic* | *p* |
| Intercept | 2.99 | 2.38 – 3.60 | 9.58 | **<0.001** | 3.19 | 2.58 – 3.80 | 10.21 | **<0.001** |
| DMN A+C & VAN B | -0.93 | -1.66 – -0.21 | -2.52 | **0.012** |  |  |  |  |
| Age | -0.03 | -0.28 – 0.22 | -0.23 | 0.815 | 0.05 | -0.28 – 0.38 | 0.31 | 0.754 |
| Education | -0.12 | -0.24 – 0.01 | -1.80 | 0.072 | -0.16 | -0.29 – -0.02 | -2.27 | **0.023** |
| Motion RMSD | 1.74 | -1.46 – 4.94 | 1.07 | 0.286 | -2.51 | -5.39 – 0.36 | -1.72 | 0.086 |
| Age * DMN A+C & VAN B | 2.01 | 0.89 – 3.13 | 3.51 | **<0.001** |  |  |  |  |
| VAN B & DAN A |  |  |  |  | -0.74 | -1.91 – 0.43 | -1.25 | 0.213 |
| Age * VAN B & DAN A |  |  |  |  | -3.43 | -5.20 – -1.65 | -3.78 | **<0.001** |
| **Random Effects** | | | | | | | | |
| σ^2^ | 3.29 | | | | 3.29 | | | |
| τ_00_ | 0.18 _sub_ | | | | 0.19 _sub_ | | | |
|  | 1.71 _Category_ | | | | 1.71 _Category_ | | | |
| ICC | 0.37 | | | | 0.37 | | | |
| Observations | 9837 | | | | 9837 | | | |
| Marginal R^2^ / Conditional R^2^ | 0.012 / 0.373 | | | | 0.013 / 0.374 | | | |

*Note*. Significant effects are marked in bold. Contrasts are sum coded. P-values were obtained via likelihood ratio tests. CI Confidence interval.

Table S5. Results for significant effects of cPPI connectivity for response time

|  | **Response time** | | | | **Response time** | | | | **Response time** | | | | **Response time** | | | |
| --- | --- | --- | --- | --- | --- | --- | --- | --- | --- | --- | --- | --- | --- | --- | --- | --- |
| *Coefficient* | *Estimates* | *CI* | *Statistic* | *p* | *Estimates* | *CI* | *Statistic* | *p* | *Estimates* | *CI* | *Statistic* | *p* | *Estimates* | *CI* | *Statistic* | *p* |
| Intercept | 6.48 | 6.44 – 6.52 | 324.58 | **<0.001** | 6.48 | 6.44 – 6.53 | 297.98 | **<0.001** | 6.53 | 6.49 – 6.57 | 302.80 | **<0.001** | 6.52 | 6.47 – 6.56 | 302.21 | **<0.001** |
| DMN A & SEM | -0.22 | -0.30 – -0.14 | -5.41 | **<0.001** |  |  |  |  |  |  |  |  |  |  |  |  |
| Age | 0.07 | 0.05 – 0.10 | 5.40 | **<0.001** | 0.07 | 0.04 – 0.10 | 4.82 | **<0.001** | 0.04 | 0.02 – 0.07 | 3.15 | **0.002** | 0.12 | 0.09 – 0.16 | 7.05 | **<0.001** |
| Education | 0.01 | -0.00 – 0.02 | 1.25 | 0.211 | -0.00 | -0.01 – 0.01 | -0.08 | 0.935 | 0.00 | -0.01 – 0.01 | 0.28 | 0.783 | 0.00 | -0.01 – 0.01 | 0.03 | 0.978 |
| Motion RMSD | 0.40 | 0.14 – 0.65 | 3.02 | **0.003** | 0.24 | -0.01 – 0.49 | 1.87 | 0.061 | 0.33 | 0.08 – 0.58 | 2.60 | **0.009** | -0.29 | -0.56 – -0.01 | -2.05 | **0.041** |
| Age * DMN A & SEM | 0.69 | 0.55 – 0.83 | 9.54 | **<0.001** |  |  |  |  |  |  |  |  |  |  |  |  |
| DMN B & DAN A |  |  |  |  | -0.23 | -0.31 – -0.14 | -5.42 | **<0.001** |  |  |  |  |  |  |  |  |
| Age * DMN B & DAN A |  |  |  |  | 0.56 | 0.41 – 0.72 | 7.21 | **<0.001** |  |  |  |  |  |  |  |  |
| SEM & VAN B |  |  |  |  |  |  |  |  | 0.02 | -0.05 – 0.09 | 0.47 | 0.642 |  |  |  |  |
| Age * SEM & VAN B |  |  |  |  |  |  |  |  | -0.57 | -0.79 – -0.35 | -5.12 | **<0.001** |  |  |  |  |
| VAN B & DAN A |  |  |  |  |  |  |  |  |  |  |  |  | -0.48 | -0.60 – -0.35 | -7.62 | **<0.001** |
| Age * VAN B & DAN A |  |  |  |  |  |  |  |  |  |  |  |  | -0.44 | -0.60 – -0.28 | -5.37 | **<0.001** |
| **Random Effects** | | | | | | | | | | | | | | | | |
| σ^2^ | 0.11 | | | | 0.11 | | | | 0.11 | | | | 0.11 | | | |
| τ_00_ | 0.01 _sub_ | | | | 0.01 _sub_ | | | | 0.01 _sub_ | | | | 0.01 _sub_ | | | |
|  | 0.00 _Category_ | | | | 0.00 _Category_ | | | | 0.00 _Category_ | | | | 0.00 _Category_ | | | |
| ICC | 0.08 | | | | 0.10 | | | | 0.09 | | | | 0.10 | | | |
| Observations | 9069 | | | | 9069 | | | | 9069 | | | | 9069 | | | |
| Marginal R^2^ / Conditional R^2^ | 0.032 / 0.113 | | | | 0.020 / 0.118 | | | | 0.020 / 0.113 | | | | 0.042 / 0.138 | | | |

*Note*. Significant effects are marked in bold. Contrasts are sum coded. P-values were obtained via likelihood ratio tests. CI Confidence interval.

Table S6. Results for linear mixed-effects model on the effect of age on brain system segregation

|  | **Brain system segregation** | | | |
| --- | --- | --- | --- | --- |
| *Coefficient* | *Estimates* | *CI* | *Statistic* | *p* |
| Intercept | 0.48 | 0.45 – 0.51 | 31.57 | **<0.001** |
| Age | 0.08 | 0.04 – 0.13 | 3.68 | **0.001** |
| Motion RMSD | -0.76 | -1.20 – -0.33 | -3.52 | **0.001** |
| **Random Effects** | | | | |
| σ^2^ | 0.00 | | | |
| τ_00_ _sub_ | 0.00 | | | |
| ICC | 0.06 | | | |
| Observations | 58 | | | |
| Marginal R^2^ / Conditional R^2^ | 0.523 / 0.553 | | | |

*Note*. Significant effects are marked in bold. Contrasts are sum coded. P-values were obtained via likelihood ratio tests. CI Confidence interval.

Table S7**.** Results for mixed-effects models on the effect of brain system segregation on accuracy and response time

|  | **Accuracy** | | | | **Response time** | | | |
| --- | --- | --- | --- | --- | --- | --- | --- | --- |
| *Coefficient* | *Log-Odds* | *CI* | *Statistic* | *p* | *Estimates* | *CI* | *Statistic* | *p* |
| Intercept | 2.97 | 2.35 – 3.58 | 9.47 | **<0.001** | 6.55 | 6.49 – 6.60 | 242.95 | **<0.001** |
| Global segregation | 2.62 | 0.64 – 4.61 | 2.59 | **0.010** | -1.31 | -1.54 – -1.08 | -11.29 | **<0.001** |
| Age | 0.08 | -0.19 – 0.35 | 0.58 | 0.560 | -0.02 | -0.05 – 0.01 | -1.57 | 0.116 |
| Education | -0.14 | -0.28 – -0.01 | -2.08 | **0.038** | 0.02 | 0.01 – 0.04 | 3.50 | **<0.001** |
| Motion RMSD | 0.83 | -2.15 – 3.81 | 0.55 | 0.584 | -1.07 | -1.41 – -0.73 | -6.17 | **<0.001** |
| Age * Global Segregation | -5.01 | -8.17 – -1.85 | -3.11 | **0.002** | 1.48 | 1.13 – 1.83 | 8.26 | **<0.001** |
| **Random Effects** | | | | | | | | |
| σ^2^ | 3.29 | | | | 0.11 | | | |
| τ_00_ | 0.17 _sub_ | | | | 0.02 _sub_ | | | |
|  | 1.71 _Category_ | | | | 0.00 _Category_ | | | |
| ICC | 0.36 | | | | 0.15 | | | |
| Observations | 9837 | | | | 9069 | | | |
| Marginal R^2^ / Conditional R^2^ | 0.010 / 0.370 | | | | 0.050 / 0.193 | | | |

*Note*. Significant effects are marked in bold. Contrasts are sum coded. P-values were obtained via likelihood ratio tests. CI Confidence interval.

Table S8**.** Results for linear mixed-effects model on the effect of age on global efficiency

|  | **Global efficiency** | | | |
| --- | --- | --- | --- | --- |
| *Coefficient* | *Estimates* | *CI* | *Statistic* | *p* |
| Intercept | 0.10 | 0.10 – 0.11 | 38.20 | **<0.001** |
| Age | 0.02 | 0.01 – 0.03 | 5.02 | **<0.001** |
| Motion RMSD | -0.08 | -0.16 – -0.00 | -2.11 | **0.040** |
| **Random Effects** | | | | |
| σ^2^ | 0.00 | | | |
| τ_00_ _sub_ | 0.00 | | | |
| ICC | 0.16 | | | |
| Observations | 58 | | | |
| Marginal R^2^ / Conditional R^2^ | 0.511 / 0.589 | | | |

*Note*. Significant effects are marked in bold. Contrasts are sum coded. P-values were obtained via likelihood ratio tests. CI Confidence interval.

Table S9**.** Results for mixed-effects models on the effect of global efficiency on accuracy and response time

|  | **Accuracy** | | | | **Response time** | | | |
| --- | --- | --- | --- | --- | --- | --- | --- | --- |
| *Coefficient* | *Log-Odds* | *CI* | *Statistic* | *p* | *Estimates* | *CI* | *Statistic* | *p* |
| Intercept | 3.05 | 2.42 – 3.67 | 9.54 | **<0.001** | 6.47 | 6.43 – 6.52 | 296.92 | **<0.001** |
| Global efficiency | 13.53 | 4.33 – 22.73 | 2.88 | **0.004** | 0.55 | -0.31 – 1.41 | 1.25 | 0.212 |
| Age | 0.13 | -0.17 – 0.43 | 0.86 | 0.387 | 0.04 | 0.01 – 0.07 | 2.85 | **0.004** |
| Education | -0.10 | -0.23 – 0.04 | -1.42 | 0.157 | 0.01 | -0.01 – 0.02 | 1.05 | 0.293 |
| Motion RMSD | 0.96 | -1.93 – 3.85 | 0.65 | 0.517 | 0.60 | 0.32 – 0.88 | 4.19 | **<0.001** |
| Age * Global efficiency | -13.71 | -33.96 – 6.54 | -1.33 | 0.185 | -5.50 | -7.23 – -3.76 | -6.21 | **<0.001** |
| **Random Effects** | | | | | | | | |
| σ^2^ | 3.29 | | | | 0.11 | | | |
| τ_00_ | 0.24 _sub_ | | | | 0.01 _sub_ | | | |
|  | 1.71 _Category_ | | | | 0.00 _Category_ | | | |
| ICC | 0.37 | | | | 0.10 | | | |
| Observations | 9837 | | | | 9069 | | | |
| Marginal R^2^ / Conditional R^2^ | 0.009 / 0.378 | | | | 0.018 / 0.112 | | | |

*Note*. Significant effects are marked in bold. Contrasts are sum coded. P-values were obtained via likelihood ratio tests. CI Confidence interval.

Table S10**.** Results for linear mixed-effects model on the effect of age on brain segregation as a function of network type

|  | **Network segregation** | | | |
| --- | --- | --- | --- | --- |
| *Coefficient* | *Estimates* | *CI* | *Statistic* | *p* |
| Intercept | 0.42 | 0.38 – 0.45 | 23.69 | **<0.001** |
| Age | -0.11 | -0.13 – -0.08 | -7.09 | **<0.001** |
| DMN A+C | 0.22 | 0.18 – 0.26 | 10.67 | **<0.001** |
| DMN B | 0.06 | 0.02 – 0.10 | 2.82 | **0.005** |
| SEM | 0.02 | -0.02 – 0.06 | 0.99 | 0.323 |
| CONT B | 0.13 | 0.09 – 0.17 | 6.43 | **<0.001** |
| VAN B | 0.17 | 0.13 – 0.21 | 8.30 | **<0.001** |
| DAN A | 0.31 | 0.27 – 0.35 | 14.86 | **<0.001** |
| Motion RMSD | -0.67 | -0.98 – -0.36 | -4.24 | **<0.001** |
| **Random Effects** | | | | |
| σ^2^ | 0.01 | | | |
| τ_00_ _sub_ | 0.00 | | | |
| ICC | 0.10 | | | |
| Observations | 406 | | | |
| Marginal R^2^ / Conditional R^2^ | 0.552 / 0.599 | | | |

*Note*. Significant effects are marked in bold. Contrasts are sum coded. P-values were obtained via likelihood ratio tests. CI Confidence interval.

Table S11**.** Results for mixed-effects models on the effect of network segregation on accuracy and response time

|  | **Accuracy** | | | | **Response time** | | | |
| --- | --- | --- | --- | --- | --- | --- | --- | --- |
| *Coefficient* | *Log-Odds* | *CI* | *Statistic* | *p* | *Estimates* | *CI* | *Statistic* | *p* |
| Intercept | 2.93 | 2.32 – 3.54 | 9.37 | **<0.001** | 6.55 | 6.47 – 6.63 | 159.97 | **<0.001** |
| DMN A | 0.49 | -0.27 – 1.26 | 1.26 | 0.208 | 0.62 | 0.52 – 0.72 | 12.02 | **<0.001** |
| DMN A+C | -0.28 | -1.67 – 1.12 | -0.39 | 0.698 | -0.24 | -0.45 – -0.02 | -2.12 | **0.034** |
| DMN B | 1.01 | -0.18 – 2.20 | 1.67 | 0.095 | 0.21 | 0.04 – 0.38 | 2.41 | **0.016** |
| SEM | -0.24 | -1.19 – 0.71 | -0.50 | 0.619 | -0.59 | -0.71 – -0.46 | -9.17 | **<0.001** |
| CONT B | 1.75 | 0.15 – 3.34 | 2.15 | **0.032** | -0.41 | -0.67 – -0.15 | -3.11 | **0.002** |
| VAN B | 1.15 | 0.06 – 2.24 | 2.07 | **0.038** | -0.16 | -0.31 – 0.00 | -1.96 | 0.050 |
| DAN A | -2.20 | -3.46 – -0.94 | -3.43 | **0.001** | -0.31 | -0.45 – -0.18 | -4.49 | **<0.001** |
| Age | -0.16 | -0.52 – 0.20 | -0.87 | 0.384 | -0.10 | -0.16 – -0.05 | -3.60 | **<0.001** |
| Education | -0.10 | -0.24 – 0.04 | -1.42 | 0.154 | -0.02 | -0.04 – -0.00 | -2.45 | **0.014** |
| Motion RMSD | 4.34 | 0.87 – 7.81 | 2.45 | **0.014** | -0.38 | -0.90 – 0.13 | -1.46 | 0.145 |
| Age * DMN A | 1.35 | -0.10 – 2.80 | 1.82 | 0.068 | -0.82 | -1.01 – -0.64 | -8.70 | **<0.001** |
| Age * DMN A+C | -1.00 | -3.66 – 1.67 | -0.73 | 0.463 | -0.29 | -0.68 – 0.09 | -1.50 | 0.133 |
| Age * DMN B | -2.80 | -5.17 – -0.42 | -2.31 | **0.021** | 0.18 | -0.15 – 0.51 | 1.07 | 0.284 |
| Age * SEM | 2.07 | -0.29 – 4.44 | 1.72 | 0.086 | 0.37 | -0.11 – 0.85 | 1.49 | 0.136 |
| Age * CONT B | 1.89 | -0.78 – 4.57 | 1.39 | 0.165 | 0.73 | 0.38 – 1.07 | 4.11 | **<0.001** |
| Age * VAN B | -5.00 | -7.22 – -2.77 | -4.40 | **<0.001** | -0.13 | -0.45 – 0.19 | -0.80 | 0.425 |
| Age * DAN A | -0.28 | -3.02 – 2.46 | -0.20 | 0.843 | 1.79 | 1.40 – 2.19 | 8.95 | **<0.001** |
| **Random Effects** | | | | | | | | |
| σ^2^ | 3.29 | | | | 0.10 | | | |
| τ_00_ | 0.07 _sub_ | | | | 0.04 _sub_ | | | |
|  | 1.71 _Category_ | | | | 0.00 _Category_ | | | |
| ICC | 0.35 | | | | 0.30 | | | |
| Observations | 9837 | | | | 9069 | | | |
| Marginal R^2^ / Conditional R^2^ | 0.033 / 0.372 | | | | 0.183 / 0.430 | | | |

*Note*. Significant effects are marked in bold. Contrasts are sum coded. P-values were obtained via likelihood ratio tests. CI Confidence interval.

Table S12**.** Connector hubs in older adults

| **Network** | **Region of interest** | **Mean PC** |
| --- | --- | --- |
| DMN A | Lateral occipital cortex, superior division 1 | 0.55 |
| DMN A | Angular gyrus 3 | 0.55 |
| DMN A+C | Lateral occipital cortex, superior division 1 | 0.54 |
| DMN A+C | Lateral occipital cortex, superior division 5 | 0.59 |
| DMN B | Frontal pole 2 | 0.54 |
| DMN B | Angular gyrus | 0.55 |
| DMN B | Middle temporal gyrus, posterior division 2 | 0.55 |
| SEM | Superior frontal gyrus 1 | 0.54 |
| SEM | Paracingulate gyrus 2 | 0.56 |
| CONT B | Frontal pole 1 | 0.54 |
| CONT B | Frontal pole 3 | 0.55 |
| CONT B | Middle frontal gyrus 3 | 0.55 |
| CONT B | Angular gyrus 1 | 0.56 |
| CONT B | Angular gyrus 2 | 0.62 |
| CONT B | Superior frontal gyrus 1 | 0.54 |
| CONT B | Paracingulate gyrus | 0.57 |
| CONT B | Frontal pole 6 | 0.54 |

*Note.* PC participation coefficient.

Table S13**.** Connector hubs in young adults

| **Network** | **Region of interest** | **Mean PC** |
| --- | --- | --- |
| DMN A | Lateral occipital cortex, superior division 1 | 0.64 |
| DMN A | Angular gyrus 3 | 0.63 |
| DMN A+C | Lateral occipital cortex, superior division 1 | 0.53 |
| DMN B | Angular gyrus | 0.58 |
| DMN B | Middle temporal gyrus, posterior division 4 | 0.52 |
| SEM | Paracingulate gyrus 2 | 0.53 |
| CONT B | Middle frontal gyrus 3 | 0.52 |
| CONT B | Angular gyrus 1 | 0.59 |
| CONT B | Angular gyrus 2 | 0.52 |
| CONT B | Lateral occipital cortex, superior division 2 | 0.58 |

*Note.* PC participation coefficient.

Table S14**.** Significant age differences in participation coefficient

| **Network** | **ROI** | **Term** | **Estimate** | **SE** | **t-value** | **p-value** | **p corr** |
| --- | --- | --- | --- | --- | --- | --- | --- |
| CONT B | Frontal pole 3 | YA | -0.13 | 0.03 | -3.70 | < 0.001 | 0.049 |
| CONT B | Paracingulate gyrus | YA | -0.10 | 0.03 | -3.75 | < 0.001 | 0.043 |
| SEM | Superior temporal gyrus | YA | -0.17 | 0.04 | -4.69 | < 0.001 | 0.002 |
| DAN A | Fusiform cortex 1 | YA | -0.20 | 0.04 | -4.87 | < 0.001 | 0.001 |
| DAN A | Fusiform cortex 2 | YA | -0.18 | 0.04 | -4.05 | < 0.001 | 0.016 |

*Note.* SE: standard error, p corr: p-value after correction for familywise error with Bonferroni-Holm method
